# A Deep Network Incorporating Depthwise Separable Convolutions for Pathological Diagnosis of Chest X‐Ray Images: A Model Development and Validation Study

**DOI:** 10.1002/hsr2.71647

**Published:** 2025-12-25

**Authors:** Na Zhang, Guanghong Deng, Wenlong Jing, Yong Li, Li Li

**Affiliations:** ^1^ Department of Emergency Medicine Sun Yat‐sen Memorial Hospital of Sun Yat‐sen University Guangzhou P.R. China; ^2^ Guangzhou Gaoke Communication Technology Co., Ltd Guangzhou P. R. China; ^3^ Guangdong Province Engineering Laboratory for Geographic Spatiotemporal Big Data, Key Laboratory of Guangdong for Utilization of Remote Sensing and Geographical Information System, Guangdong Open Laboratory of Geospatial Information Technology and Application, Guangzhou Institute of Geography Guangdong Academy of Sciences Guangzhou P. R. China

**Keywords:** chest X‐ray images, deep learning, dense block, pathological diagnosis

## Abstract

**Background:**

Pathological diagnosis of chest X‐ray images has always been a very challenging subject.

**Methods:**

We propose a chest X‐ray pathology detection network that fuses two depthwise separable convolutions (TDCheXNet) to detect pathology from chest X‐ray images. We remove the transformation layer used by the original CheXNet and embed it in depthwise separable convolutions for down‐sampling. In the first layer of convolution, to extract more pathological information, we use another depth‐separable convolution to replace it and make full use of the negative X‐axis features in the corresponding convolution layer.

**Results:**

The ChestX‐ray14 public chest X‐ray dataset is used, which contains more than 100,000 frontal X‐ray images covering 14 diseases. Using the area under the receiver operating characteristic curve (AUROC) as the evaluation index, calculate the average AUROC (AVG_AUROC). Experimental results show that TDCheXNet achieved 82.8% on the test set and the detection speed reached 178.794 ms, compared with the original model, AVG_AUROC is improved by 0.5%, and the single‐image inference speed is increased by 14.946 ms.

**Conclusion:**

We propose a new network for chest X‐ray pathology detection, and experimental results show that it can achieve better performance on public datasets.

## 1 Introduction

Chest X‐ray images diagnosis [1], which is an essential skill for radiologists, is a complex problem. It is mainly used to diagnose diseases of the chest, chest contents and nearby structures. Chest X‐ray is a projection radiograph of the chest produced by a small dose of ionizing radiation, imaging the density of subtle lesions and human organs, and performing pathological analysis and prediction based on the imaging results. Not only does it require careful observation, but it also requires a solid foundation in physiology and pathology. How to use computer vision methods to achieve automatic diagnosis of chest X‐ray images is an extremely challenging task. There are many types of chest diseases, which undoubtedly adds many difficulties to the diagnosis of artificial intelligence. In view of the serious shortage of medical resources and the excessive requirements for medical image analysis, how to develop an artificial intelligence system that can not only efficiently diagnose but also accurately predict various diseases has been put on the agenda.

In recent years, with the development of deep convolutional neural networks [2, 3], deep convolutional models [4, 5] can automatically and effectively extract representative features in data and enhance the performance of target data. Some diagnostic techniques based on image classification have adopted deep learning models. They conduct refined designs on original classification networks such as VGG [6], ResNet [7] and DenseNet [8]. Chest X‐ray examination is an extremely important field in the diagnosis of medical lesions, and many experts and scholars have done more research in this field [9]. Applications based on deep learning methods have also been greatly improved. Roof et al. [10] explain many of the methods available for chest X‐rays and analyze the limitations in this field. As early as the 1960s, the advent of automatic detection systems for chest X‐ray image lesions made great progress in this research field [11, 12, 13], especially in situations where radiologists could not achieve it manually [14, 15]. With the development of automatic diagnosis technology, the application of deep learning technology in the field of medical imaging has had a huge impact [16]. A well‐performing deep learning model requires a large amount of labeled data sets for training. At present, many experts and scholars have produced many data sets for research [17, 18, 19, 20], which has once again made great progress in deep learning models. van Ginneken et al. [21] explain how machine learning has become the dominant technology for auxiliary chest diagnosis and illustrate the main differences between traditional methods, machine learning and deep learning [22, 23]. The application of an efficient chest X‐ray system not only uses deep learning technology, but also relies on computer‐aided diagnosis technology [24, 25]. Baltruschat et al. [26] studied two advanced image preprocessing techniques, using bone suppression and automatic lung field detection, to improve chest X‐ray disease classification. Shin et al. [27] proposed a deep learning model to effectively detect diseases from chest X‐ray images and annotate information such as location, severity, and affected organs. Cai et al. [28] proposed an attention mining method for disease localization, which showed good results in chest X‐ray pathological diagnosis. Wang et al. [29] proposed a novel text‐image embedding network for extracting image and text representation of chest X‐rays. HydraViT [30] effectively integrates the Transformer module with convolutional neural network feature extraction to improve the accuracy of multi‐label classification. Focal modulation network [31] uses global and local attention mechanisms to jointly model the contextual relationship of chest X‐ray images. An end‐to‐end convolutional neural network [32] based on an attention mechanism can effectively retrieve similar images in large X‐ray datasets. In summary, the above research on chest X‐ray pathology detection has low detection accuracy and slow detection reasoning speed, which can no longer meet today's needs. Artificial intelligence‐assisted chest X‐ray diagnostic methods often require higher detection accuracy and faster detection speed. Existing algorithms currently cannot meet the requirements of real radiologists for chest X‐ray detection.

The methods with many appeals no longer meet the requirements for chest X‐ray diagnosis in real life. The ChestX‐ray14 [17, 33] is an open‐source chest radiograph data set. The dataset contains 14 common chest pathologies, including atelectasis, Cardiomegaly, Effusion, Infiltration, Mass, Nodule, Pneumonia, Pneumothorax, Consolidation, Edema, Emphysema, Fibrosis, Pleural Thickening and Hernia. It is a large‐scale dataset that is suitable for experiments. This article improves the original CheXNet [34] model for chest X‐ray diagnosis. We remove the original transformation layer and use depthwise separable convolutions (DSC) [35] for down‐sampling. To make more use of the convolution feature map information, the first‐layer convolution machine is improved and uses separable convolution with a convolution kernel of the same size as the original, so that more effective information can be fully utilized for the first input of the original image. At the same time, we have made more use of the input information in the negative direction of the x‐axis after convolution extraction. We named this network TDCheXNet. Existing research addresses the inability of deep networks to achieve high accuracy and fast inference speed. While deep networks have been used for pathology diagnosis, they still fall short of the rapid diagnosis required by radiologists. We have improved the inference speed of chest X‐ray pathology diagnosis by embedding the DSC module within the deep network model, effectively increasing inference speed.

Therefore, the Main Contributions of This Article Are As Follows

1. We propose a novel TDCheXNet model that uses deep neural networks and chest X‐ray diagnosis to improve the ability of pathological radiographic diagnosis.
2. We propose an improved transformation layer to enhance the region of interest features of chest X‐ray images during down sampling.
3. We use separable convolutions of the same size as the first‐layer convolution kernel of the original network to make the application of feature maps more effective.
4. We make full use of the information in the negative direction of the x‐axis of the feature map extracted by convolution to improve the robustness of the model.

## 2 Methods

**Figure 1:**
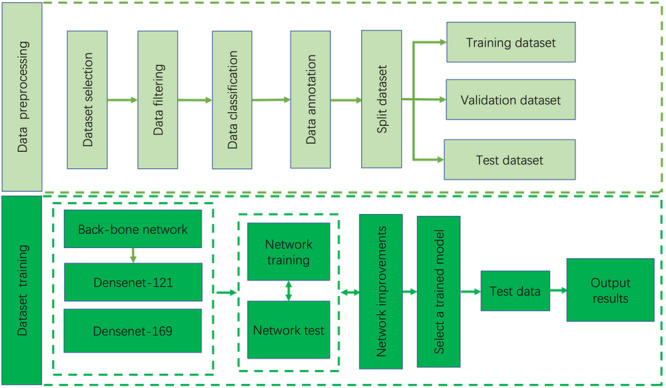
Model training flow chart.

We improved the original CheXnet and proposed a new network model for chest X‐ray pathology classification. The main implementation steps are divided into two parts: data processing and dataset training and tuning. Model training flow chart is show in Figure 1.

As shown in Figure 1, the first step is data processing. We need to select data, clean data, classify data, label data, and divide the data set into training set, test set, and validation set. Next comes the dataset training phase, where the divided dataset is fed into the model training and validation phase. Our model's backbone network is primarily composed of two models of varying depths: DenseNet‐121 and DenseNet‐169. These models are then refined, trained, and validated. Finally, the optimal model is output on the same dataset.

### 2.1 CheXNet

Using deep learning to improve the efficiency of radiologists’ chest X‐ray diagnosis has always been an extremely challenging topic. How to design an efficient deep network model is a primary problem to be solved. CheXNet is a deep convolutional neural network model mainly used for diagnosis of chest X‐ray images. The backbone network is a DenseNet‐121 model, as shown in Table 1.

**Table 1:** CheXNet network.

Layers	Output size	Densenet‐121
Convolution	112×112	
Pooling	56×56	
Dense Block (1)	56×56	1×1conv3×3conv×6
Transition Layer (1)	56×5628×28	1×1×128conv
Dense Block (2)	28×28	1×1conv3×3conv×12
Transition Layer (2)	28×2814×14	1×1×256conv
Dense Block (3)	14×14	1×1conv3×3conv×24
Transition Layer (3)	14×147×7	1×1×512conv
Dense Block (4)	7×7	1×1conv3×3conv×16
Classification Layer	14	

**Figure 2:**
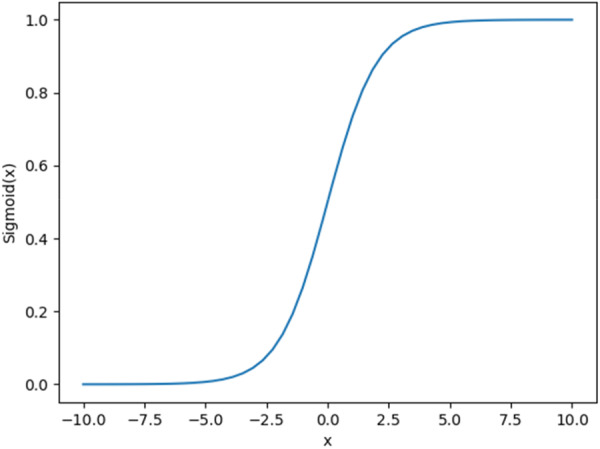
Sigmoid function.

As can be seen from Table 1, the CheXNet network mainly consists of convolutional layers, pooling layers, dense blocks, and conversion layers. The network has a total of four dense blocks. Each dense block is composed of a different number of 1×1 and 3×3 convolution blocks. The final step of the network is to replace the fully connected layer of DenseNet with a 14‐dimensional binary output and connect a Sigmoid unit [36, 37, 38, 39, 40] to output a probability value. This is a typical Sigmoid function application. The Sigmoid function is shown in Figure 2.

### 2.2 Improved CheXNet

#### 2.2.1 Improved Convolution Layer

**Figure 3:**
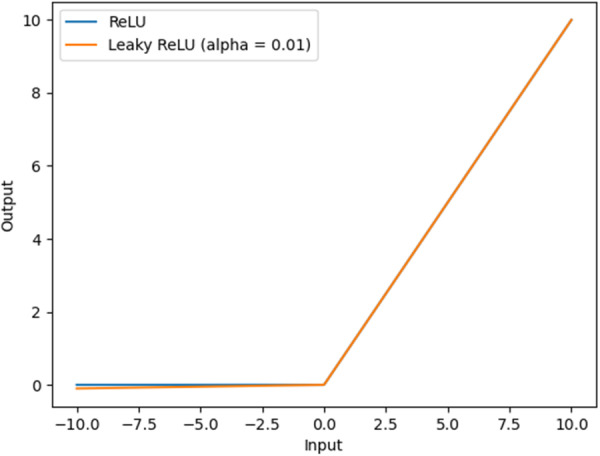
The ReLU and LReLU function curves.

Although the original CheXNet achieved good results in the field of chest X‐ray diagnosis, there is still some room for optimization to improve the model performance. CheXNet uses a convolution kernel size of 7×7 to extract features from the input original image. The convolution kernel sizes used in dense blocks are 1×1 and 3×3. The activation function uses Rectified Linear Unit (ReLU) [41, 42, 43, 44]. Although using a larger convolution kernel can cover a larger image area and obtain wider context information, it reduces the sharing of weights at different locations, which may cause a reduction in the performance of different networks. Using the ReLU function with a lower amount of calculation, there will be no problems such as gradient saturation or disappearance when the input x > 0, but when the input x < 0, the gradient of this neuron and subsequent neurons will always be 0, resulting in the corresponding parameters will not be updated. To solve this problem, we use the Leaky ReLU (LReLU) [45] function that can still respond to a negative input value. The ReLU and LReLU function curves are shown in Figure 3.

#### 2.2.2 Improved Transition Layer

**Figure 4:**
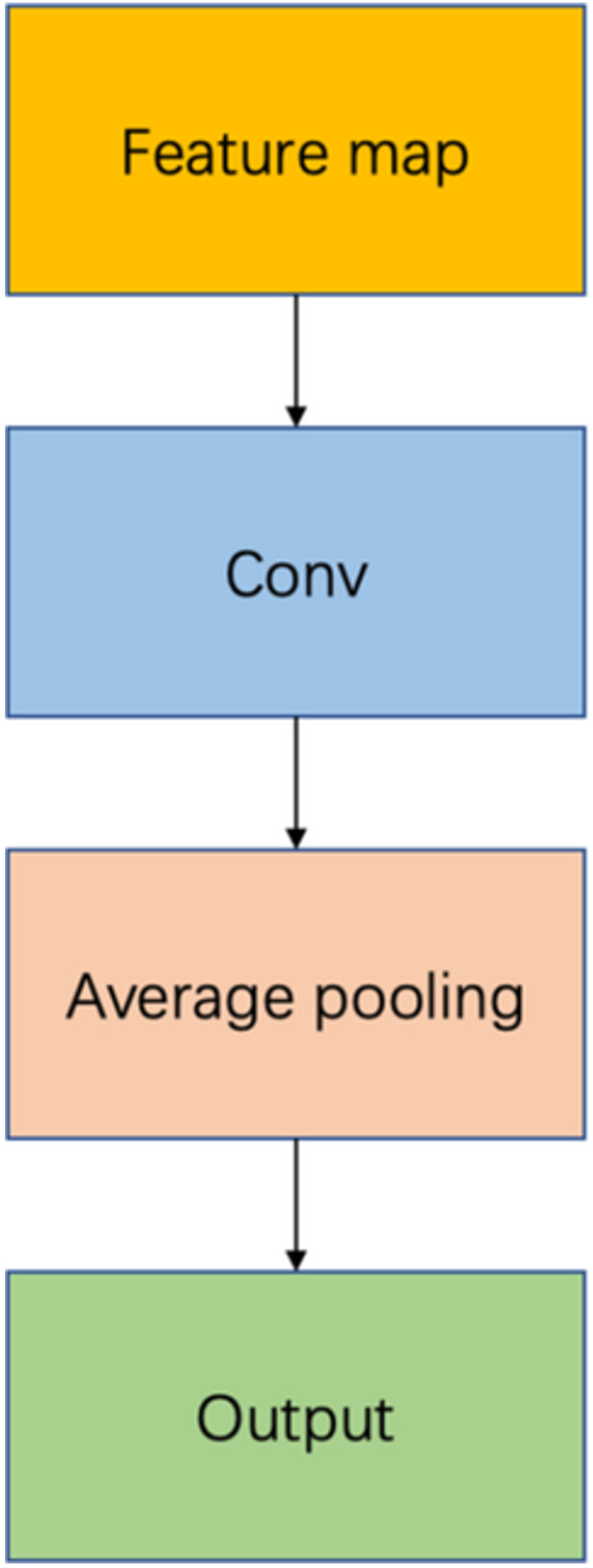
Transition layer.

The transition layer of CheXNet mainly implements changes to the size and number of channels of the input feature map and is mainly composed of a 1×1 convolution kernel and an average pooling layer with a stride of 2. The transition layer is shown in Figure 4.

Average pooling [46, 47] is a commonly used pooling operation in convolutional neural networks. The purpose is to reduce the spatial dimension of the data while retaining important feature information, which can effectively reduce the amount of calculation and the number of parameters. Average pooling is to calculate the mean of all elements in the window area for a fixed‐size window (such as 2×2) and use the mean to represent the feature information in the window. Therefore, each window is reduced to a single value, thereby achieving a dimensionality reduction effect. The advantage of average pooling is that it can smoothly integrate feature information, which plays a crucial role in some tasks that require detailed texture information. Average pooling is shown in formula (1).

(1) $$y_{\mathrm{kij}}=\frac{1}{|R_{\mathrm{ij}}|}\sum_{(p,q)\in R_{\mathrm{ij}}}x_{\mathrm{kpq}}$$

where $y_{\mathrm{kij}}$ represents the pooling output related to the kth feature map, $x_{\mathrm{kpq}}$ is the element at (p, q) in the pooling region $R_{\mathrm{ij}}$.

Average pooling performs a down‐sampling operation. Although it can retain finer‐grained information, it cannot highlight the more significant features of pathology. DSC mainly consists of two parts. First, depth convolution is used to extract features using a single convolution filter for each input channel of the feature map. Secondly, pointwise convolution is used to combine each channel. DSC and its improved models [48, 49, 50] have been verified to achieve better results when embedded in deep convolutional neural networks.

**Figure 5:**
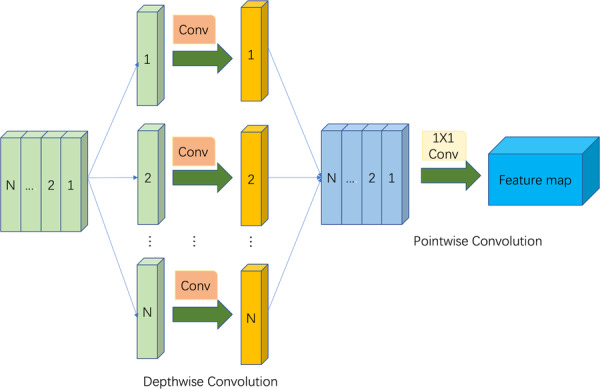
Improved conversion layer uses depthwise separable convolutions.

In the CheXNet network, shallow texture and edge information need to be learned, while salient features in depth information must also be highlighted. Therefore, we improved the original transition layer. To learn deep feature information more effectively, the DSC is introduced in the original transition layer. To reduce model parameters, we remove the convolutional layer and pooling layer of the original transition layer and directly use DSC instead. Therefore, we propose an improved transition layer. The improved transition layer is shown in Figure 5.

#### 2.2.3 TDcheXNet Network

**Figure 6:**
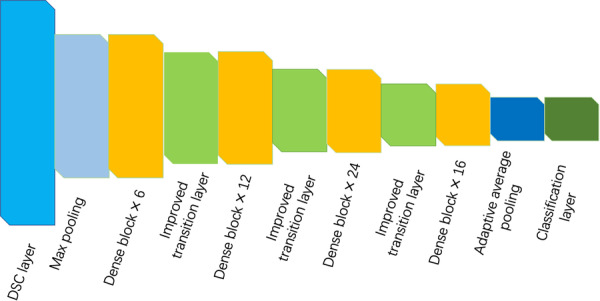
TDCheXNet Network.

Since the original CheXNet network consists of many dense blocks, the size and number of channels of the feature maps are changed through the transition layer. A deeper network structure can help improve network performance. Although improving the transition layer can prevent the loss of multi‐layer information flow, there are still problems such as the deeper network structure not fully utilizing layer‐by‐layer feature information. Therefore, we removed the first layer convolution of the original DenseNet‐121 from the backbone network and used DSC instead. We named this chest X‐ray pathology network that combines two depthwise separable convolutions TDChXNet. The model structure is shown in Figure 6.

As can be seen from Figure 5, The first layer of the TDCheXNet network still uses DSC to extract features, and then down samples the input convolution feature map through max pooling. It has four dense modules, consisting of 6, 12, 24 and 16 dense blocks respectively. The down sampling operation of the input feature map is implemented through an improved transition layer between each dense module. Finally, the result is output through the adaptive average pooling layer and classification layer.

TDCheXNet is a deep convolutional neural network, and the network forward propagation algorithm of the network is shown in Table 2.

**Table 2:** Forward propagation algorithm of TDCheXNet network.

Algorithm 1:	TDCheXNet
Input:	Raw chest X‐ray image I_raw
Output:	Disease probabilities Y
1.	I ← Resize (I_raw, target_size)
2.	X ← DSCConv(I)//DSC layer
3.	X ← MaxPooling(X)
4.	X ← DenseBlock(X, layers=6)
5.	X ← ImprovedTransition(X)
6.	X ← DenseBlock(X, layers=12)
7.	X ← ImprovedTransition(X)
8.	X ← DenseBlock(X, layers=24)
9.	X ← ImprovedTransition(X)
10.	X ← DenseBlock(X, layers=16)
11.	X ← AdativeAveragePooling(X)
12.	Y ← ClassificationLayer(X)
13.	Return Y

As shown in Table 2, the input image needs to be preprocessed first. The preprocessing method in this paper is to maintain a certain aspect ratio of the image, then randomly crop out a fixed pixel area, perform data enhancement methods such as random horizontal flipping, and finally normalize it. The image is sent to the deep convolutional network for a series of feature extraction and finally output the predicted category.

### 2.3 Pathological Display Method

To better display pathological areas, class activation mappings (CAMs) [51] is used to generate heat maps to visualize the image areas where disease is most likely to exist. To obtain CAMs, the output feature map of the last convolutional layer is extracted by feeding the image into our trained model. As shown in formula (2).

(2) $$M_{c}=\sum_{k}w_{c,k}f_{k}$$

where $f_{k}$ represents the kth feature map, $w_{c,k}$ represents the weight of the final classification layer of feature map k that leads to pathology c. $M_{c}$ represents the most significant feature map with pathology c. Finally, by enlarging Mc to the size of the image and overlaying the image, the salient features of the model in predicting pathology c are determined.

### 2.4 Evaluation Indicators

The area under the receiver operating characteristic curve (AUROC) [52, 53] is a commonly used metric for evaluating model performance of neural networks. It mainly measures the model's ability to rank positive samples before negative samples. The value of AUROC ranges from 0 to 1. The larger the value, the better the generalization performance of the model. It still has good performance in the case of unbalanced data sets. We used chest pathological analysis to test the AUROC value of each category, also calculated the average AUROC (AVG_AUROC) value as an evaluation index. As shown in formula (3).

(3) $$\mathrm{AVG\_AUROC}=\;\frac{\sum_{q=1}^{Q}\mathrm{AUROC}(q)}{Q}$$

where Q represents the number of categories, and AUROC(q) represents the AUROC value of category q.

In addition, this paper uses precision, recall, $F_{1}$ value, AP (average precision) and mAP (mean average precision) as the evaluation indicators of the model, as shown in the following table.

(4) $$P=\;\frac{\mathrm{TP}}{\mathrm{TP}+\mathrm{FP}}$$

(5) $$R=\;\frac{\mathrm{TP}}{\mathrm{TP}+\mathrm{FN}}$$

(6) $$F_{1}=\;\frac{2\mathrm{PR}}{P+R}$$

(7) $$\mathrm{AP}=\;\int_{0}^{1}P\left(R\right)\mathrm{dR}$$

(8) $$\mathrm{mAP}=\;\frac{\sum_{q=1}^{Q}\mathrm{AP}(q)}{Q}$$

where *P* is precision, R is recall. *TP* is the true positives; *FP* is the false positives; and *FN* is the false negative.

### 2.5 Ethics Statement

All experiments in this study were conducted using the public ChestX‐ray14 [17, 33] dataset. This dataset is fully anonymized and contains no personal identity information. As such, this research qualifies for exemption from ethical review, and no approval from an ethics committee was sought.

## 3 Experiment and Results

### 3.1 Experimental Conditions

#### 3.1.1 Data Set

**Figure 7:**
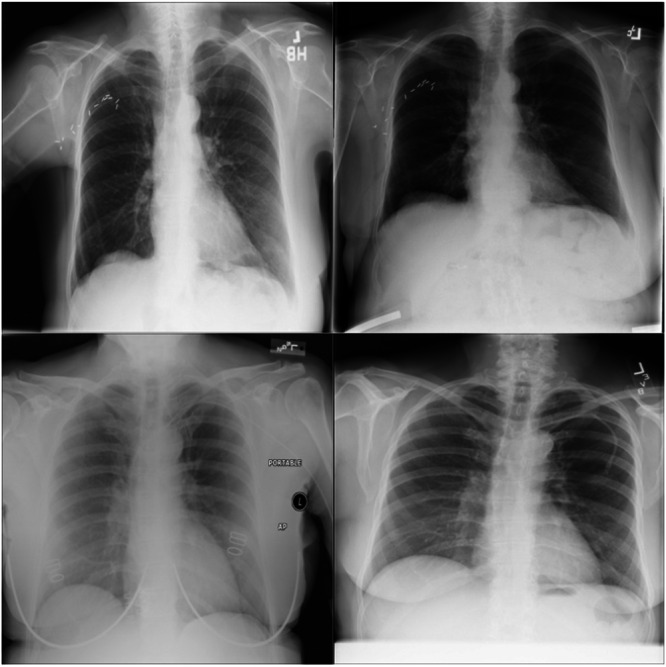
The original image.

The experimental data set in this article uses the ChestX‐ray14 open‐source chest radiograph data set. The dataset contains 14 common chest pathologies, including atelectasis, Cardiomegaly, Effusion, Infiltration, Mass, Nodule, Pneumonia, Pneumothorax, Consolidation, Edema, Emphysema, Fibrosis, Pleural Thickening and Hernia. The original images of the data set are shown in Figure 7. This dataset contains an image with one or more pathologies.

**Figure 8:**
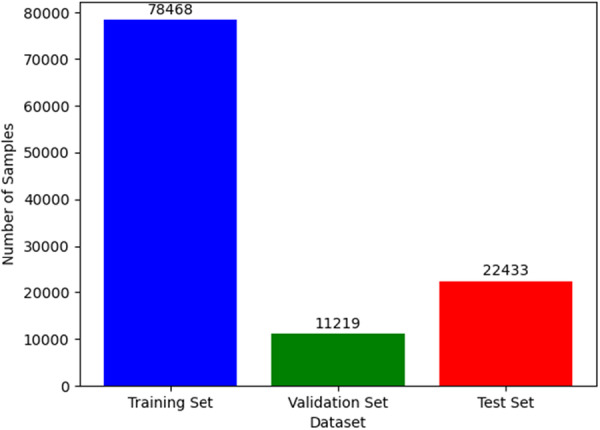
Training data set.

During the training process using this data set, we divided all data sets with a total of 112,120 images into 78,468 training sets, 11,219 verification sets, and 22,433 test sets. As shown in Figure 8.

#### 3.1.2 Experiment Platform

Server‐side: Ubuntu 20.04, Intel® Xeon® Silver 4210 R CPU@2.40 GHz, NVIDIA GeForce RTX A6000(48 GB) GPU.

### 3.2 Experimental Results

#### 3.2.1 Different Model Detection Results

To verify the effectiveness of the improved method in this article, we trained different improved models and verified them on the ChestX‐ray14 open‐source data set. The naming methods of different models are shown in Table 3.

**Table 3:** Different model naming methods.

Model	Backbone	Improved transition layer	LReLU	Improve the first layer of convolution
CheXNet	Densenet‐121			
DCheXNet	Densenet‐121	Yes		
DLCheXNet	Densenet‐121	Yes	Yes	
TDCheXNet	Densenet‐121	Yes	Yes	Yes
LCheXNet	Densenet‐169			
LDCheXNet	Densenet‐169	Yes		
LDLCheXNet	Densenet‐169	Yes	Yes	
LTDCheXNet	Densenet‐169	Yes	Yes	Yes

It can be seen from Table 3 that the original CheXNet is a chest pathology detection model with density‐121 as the backbone network. We improved the transition layer using DSC, named DCheXNet. We improved the conversion layer and linear activation using DSC, named DLCheXNet. We use DSC to improve the conversion layer, linear activation and first layer convolution, named TDCheXNet. We use denseness‐169 instead of densenet‐121 as the model for the backbone network because it is larger. Therefore, they were named LCheXNet, LDCheXNet, LDLCheXNet and LTDCheXNet.

To verify the effectiveness of the algorithm model in this article, we compared eight different chest pathology detection models. We train and validate on the same dataset respectively. For the model trained in this article, the verification results of the model with densenet‐121 as the backbone network on the test set are shown in Table 4, and the results on the validation set are shown in Table 5. The verification results of the model with densenet‐169 as the backbone network on the test set are shown in Table 6, and the results on the validation set are shown in Table 7.

**Table 4:** Compare the results on the test set results.

Pathology	CheXNet	DCheXNet	DLCheXNet	TDCheXNet
Atelectasis	0.804	0.804	0.804	0.805
Cardiomegaly	0.911	0.912	0.914	0.914
Effusion	0.882	0.881	0.881	0.882
Infiltration	0.702	0.697	0.696	0.701
Mass	0.831	0.830	0.826	0.833
Nodule	0.734	0.738	0.730	0.742
Pneumonia	0.749	0.754	0.747	0.750
Pneumothorax	0.862	0.855	0.855	0.861
Consolidation	0.804	0.801	0.803	0.802
Edema	0.889	0.889	0.892	0.895
Emphysema	0.889	0.883	0.885	0.895
Fibrosis	0.811	0.815	0.809	0.820
Pleural Thickening	0.771	0.769	0.761	0.773
Hernia	0.886	0.905	0.907	0.913
AVG_AUROC	0.823	0.824	0.822	0.828

**Table 5:** Compare the results on the validation set.

Pathology	CheXNet	DCheXNet	DLCheXNet	TDCheXNet
Atelectasis	0.798	0.798	0.798	0.804
Cardiomegaly	0.902	0.909	0.910	0.906
Effusion	0.878	0.879	0.879	0.878
Infiltration	0.695	0.693	0.690	0.693
Mass	0.866	0.861	0.863	0.862
Nodule	0.737	0.742	0.729	0.736
Pneumonia	0.756	0.764	0.766	0.758
Pneumothorax	0.877	0.877	0.871	0.879
Consolidation	0.821	0.821	0.817	0.823
Edema	0.914	0.916	0.915	0.915
Emphysema	0.858	0.854	0.847	0.859
Fibrosis	0.805	0.799	0.792	0.803
Pleural Thickening	0.793	0.791	0.787	0.791
Hernia	0.841	0.818	0.843	0.860
AVG_AUROC	0.824	0.823	0.822	0.826

**Table 6:** Compare the results on the test set results.

Pathology	LCheXNet	LDCheXNet	LDLCheXNet	LTDCheXNet
Atelectasis	0.804	0.802	0.804	0.803
Cardiomegaly	0.913	0.913	0.913	0.915
Effusion	0.882	0.880	0.880	0.881
Infiltration	0.700	0.697	0.697	0.699
Mass	0.828	0.827	0.831	0.830
Nodule	0.740	0.729	0.736	0.738
Pneumonia	0.748	0.750	0.751	0.755
Pneumothorax	0.859	0.855	0.855	0.862
Consolidation	0.803	0.801	0.800	0.802
Edema	0.892	0.889	0.891	0.892
Emphysema	0.885	0.881	0.882	0.898
Fibrosis	0.808	0.805	0.812	0.814
Pleural Thickening	0.768	0.765	0.762	0.768
Hernia	0.889	0.896	0.900	0.924
AVG_AUROC	0.823	0.821	0.822	0.827

**Table 7:** Compare the results on the validation set.

Pathology	LCheXNet	LDCheXNet	LDLCheXNet	LTDCheXNet
Atelectasis	0.798	0.796	0.801	0.798
Cardiomegaly	0.907	0.905	0.907	0.908
Effusion	0.879	0.877	0.878	0.880
Infiltration	0.693	0.692	0.691	0.694
Mass	0.857	0.859	0.861	0.860
Nodule	0.735	0.733	0.731	0.730
Pneumonia	0.764	0.757	0.751	0.755
Pneumothorax	0.875	0.875	0.873	0.879
Consolidation	0.821	0.820	0.822	0.823
Edema	0.917	0.915	0.916	0.918
Emphysema	0.852	0.847	0.851	0.859
Fibrosis	0.804	0.801	0.795	0.804
Pleural Thickening	0.793	0.782	0.786	0.790
Hernia	0.824	0.849	0.833	0.872
AVG_AUROC	0.823	0.822	0.821	0.826

It can be seen from Table 4 that the AVG_AUROC value of the original CheXNet model with Densenet‐121 as the backbone network in the test set is 0.823. The AVG_AUROC value of DCheXNet using the improved conversion layer is 0.824, which is 0.01 higher than CheXnet. This shows that using the improved conversion layer to down‐sample the deep neural network is effective in the application of feature maps. The DLCheXNet and TDCheXNet models are 0.822 and 0.828 respectively. This shows that using the first‐layer DSC model to extract pathological features in a deep convolutional network can effectively make full use of the features in the negative direction of the x‐axis and improve the detection performance of the network model. Table 5 shows the test results on the validation set. The AVG_AUROC values of CheXNet, DCheXNet, DLCheXNet and TDCheXNet are 0.824, 0.823, 0.822 and 0.826 respectively. On the validation set, TDCheXNet of this article still achieved the best results.

Tables 6 and 7 respectively show the improvements made to the model using densenet‐169 as the backbone network, and the test results on the test set and validation set respectively. From Table 6, the AVG_AUROC values of LCheXNet, LDCheXNet, LDLCheXNet and LTDCheXNet are 0.823, 0.821, 0.822 and 0.827 respectively. Among them, LTDCheXNet achieved the best results, but it was still 0.01 lower than TDCheXNet. From Table 7, the AVG_AUROC values of LCheXNet, LDCheXNet, LDLCheXNet and LTDCheXNet are 0.823, 0.822, 0.821 and 0.826 respectively. Among them, LTDCheXNet achieved the best results, compared to TDCheXNet, it achieved the same results.

mAP is an important indicator for measuring the performance of a model. The ablation experiments of different models are shown in Table 8.

**Table 8:** Ablation experiments of different models.

Backbone	Improved transition layer	LReLU	Improve the first layer of convolution	mAP
Densenet‐121				0.225
Densenet‐121	Yes			0.222
Densenet‐121	Yes	Yes		0.222
Densenet‐121	Yes	Yes	Yes	0.228
Densenet‐169				0.224
Densenet‐169	Yes			0.218
Densenet‐169	Yes	Yes		0.220
Densenet‐169	Yes	Yes	Yes	0.228

Table 8 shows that for the Densenet‐121‐based model, improving the conversion layer, LReLU, and the first convolutional layer helps improve the model's mAP performance. The final mAP for the Densenet‐169‐based model is the same.

**Figure 9:**
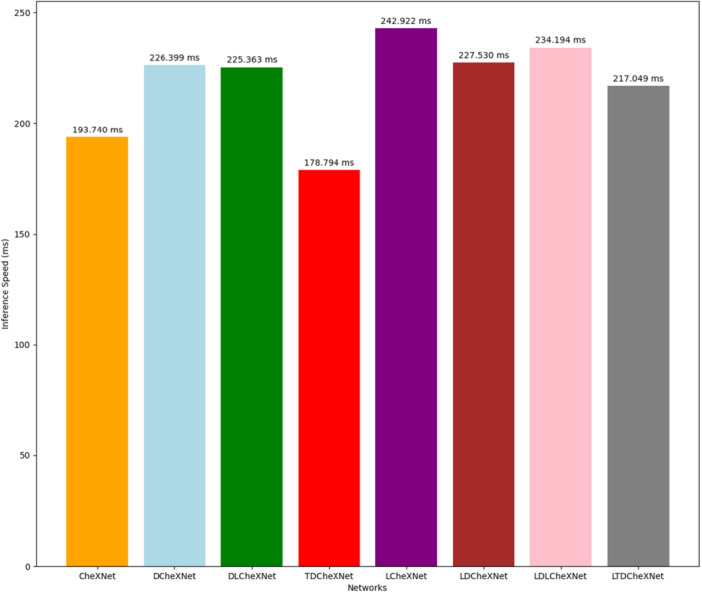
Speed comparison.

To verify the performance of a model, its detection speed is an extremely important indicator. We tested the speed comparison of different models, as shown in Figure 9.

As can be seen from Figure 9, the detection speeds of CheXNet, DCheXNet, DLCheXNet, TDCheXnet, LCheXNet, LDCheXNet, LDLCheXNet and LTDCheXNet are 193.740 ms, 226.399 ms, 225.363 ms, 178.794 ms, 242.922 ms, 227.530 ms, 234.194 ms and 217.094 ms, respectively. Therefore, the detection speed of the TDCheXNet network proposed in this article is very fast.

We trained different models on the same open‐source data set to verify whether a model has better performance capabilities. The loss function is an important indicator during the training process.

**Figure 10:**
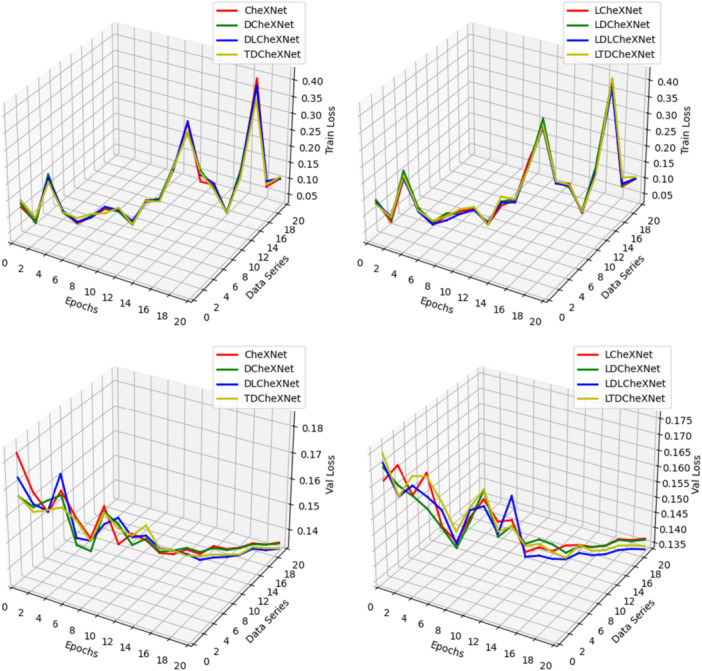
Loss function graphs of different models.

Figure 10 shows the training and validation losses of different models. We trained on the same dataset for 20 epochs. Because the loss function value is extremely small, to display our loss function image more intuitively, a 3D waterfall chart is used. The two pictures above are the training losses of different models. We can conclude that the loss values are extremely small. The following two figures show the verification losses of different models. The loss function decreases smoothly and converges to a certain extent. It shows that the training method in this article is very effective.

**Figure 11:**
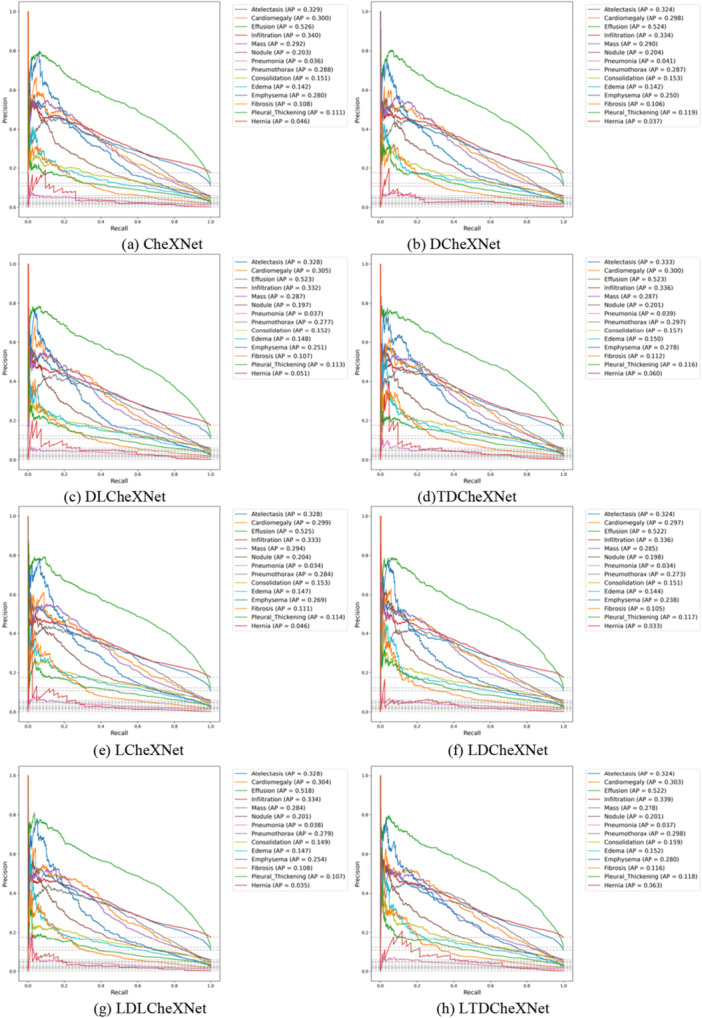
P‐R curves of different model. (a) CheXNet. (b) DCheXNet. (c) DLCheXNet. (d) TDCheXNet. (e) LCheXNet. (f) LDCheXNet. (g) LDLCheXNet. (h) LTDCheXNet.

**Figure 12:**
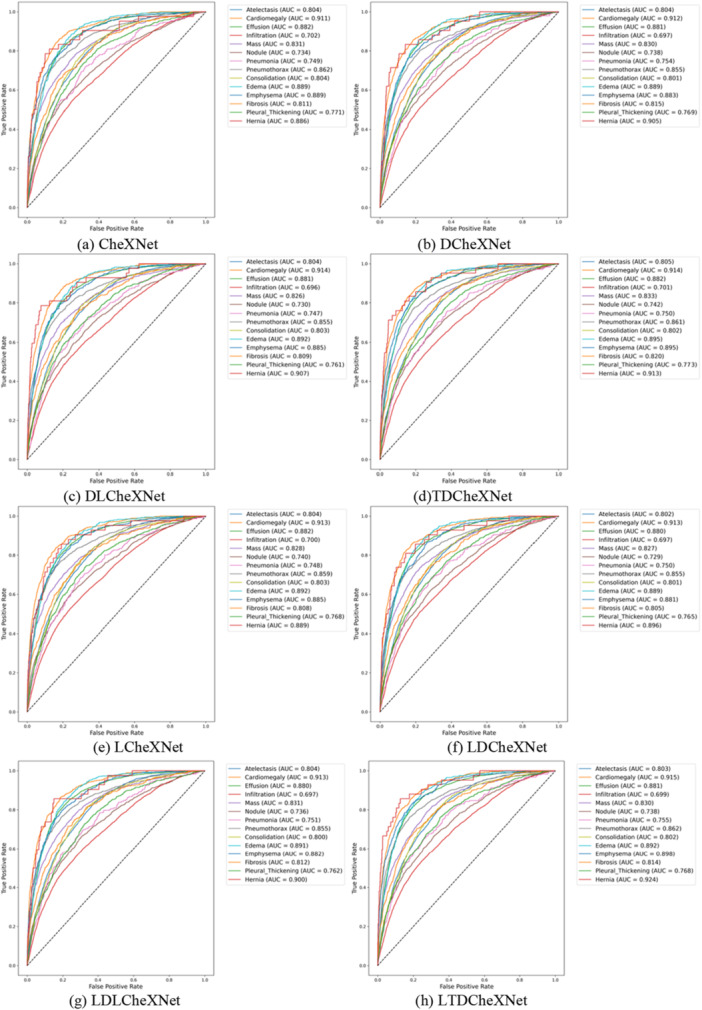
ROC curves of different models. (a) CheXNet. (b) DCheXNet. (c) DLCheXNet. (d) TDCheXNet. (e) LCheXNet. (f) LDCheXNet. (g) LDLCheXNet. (h) LTDCheXNet.

**Figure 13:**
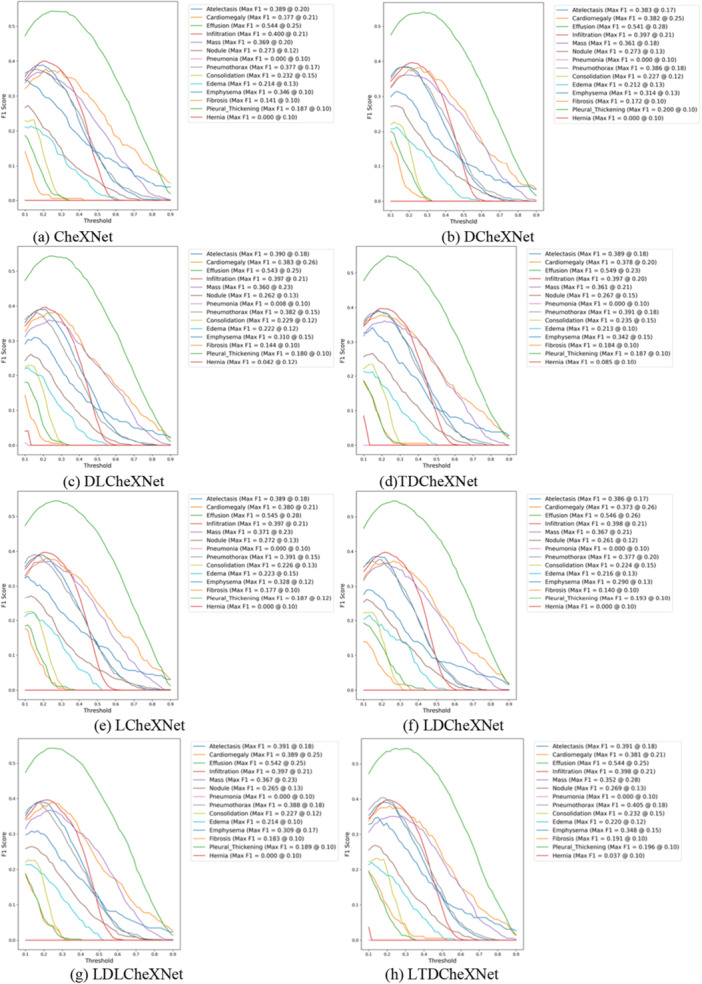
F1 curves of different models. (a) CheXNet. (b) DCheXNet. (c) DLCheXNet. (d) TDCheXNet. (e) LCheXNet. (f) LDCheXNet. (g) LDLCheXNet. (h) LTDCheXNet.

**Figure 14:**
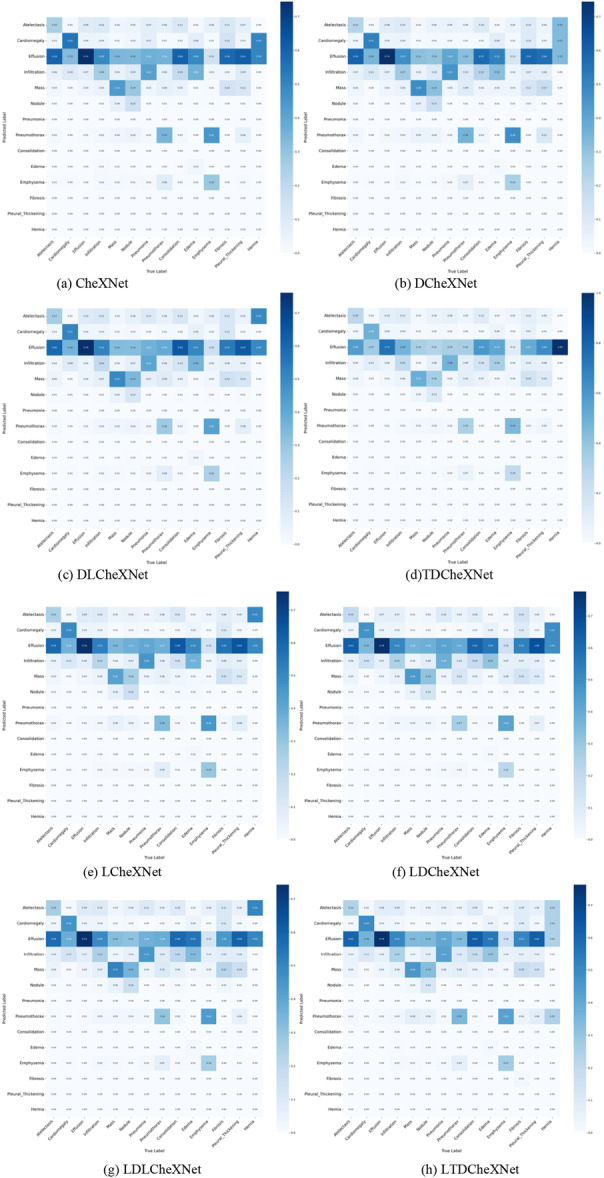
Confusion Matrix of different models. (a) CheXNet. (b) DCheXNet. (c) DLCheXNet. (d) TDCheXNet. (e) LCheXNet. (f) LDCheXNet. (g) LDLCheXNet. (h) LTDCheXNet.

The P‐R curve is an important indicator for measuring whether a model is accurate in predicting categories and whether the model performance is stable. This article trains different models and plots the P‐R curves as shown in Figure 11. The ROC curve can intuitively show whether the model performance is robust. The ROC curves of different models drawn in this article are shown in Figure 12. The F1 performance metric is a comprehensive calculation of precision and recall. The F1 performance of different models is shown in Figure 13. The confusion matrix can intuitively show the difference between the predicted category and the true label. The confusion matrix plotted in this article is shown in Figure 14.

Figure 11 shows the P‐R curve, demonstrating the effectiveness of our trained model and its ability to predict the pathology category of chest X‐ray images to a certain extent. Figure 12 shows the ROC curves for different models, demonstrating that our proposed method outperforms the original CheXnet model. Figure 13 shows the F1 performance metric for different models, demonstrating the robustness of our proposed model. Figure 14 shows the confusion matrix for different models, demonstrating that our method can reduce the false positive rate. In summary, the performance of this model is better than the performance of the CheXNet model proposed in reference [34].

#### 3.2.2 Pathological Testing Display

**Figure 15:**
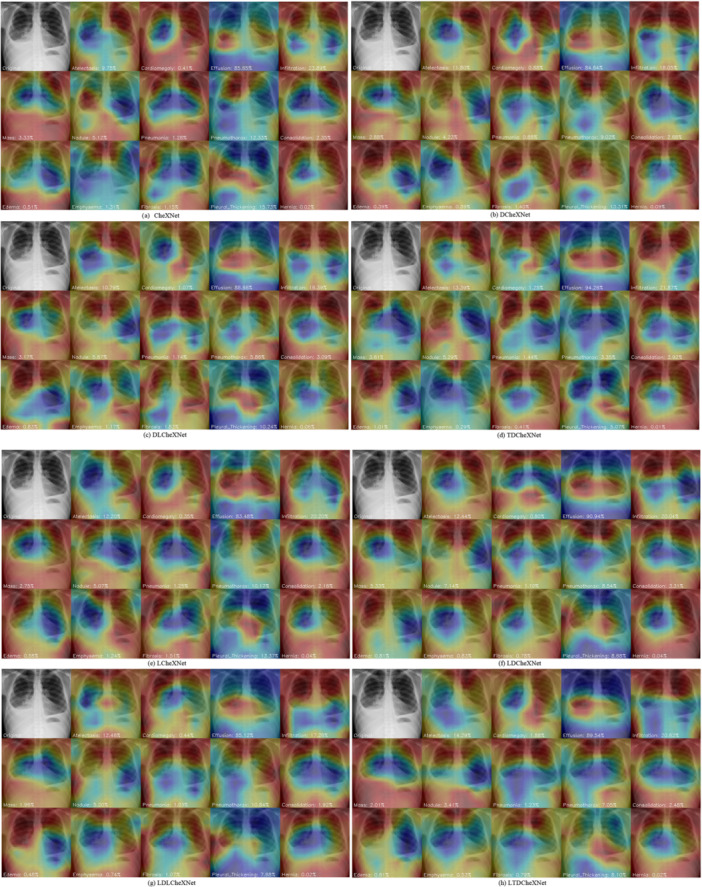
Pathological display of different models. (a) CheXNet. (b) DCheXNet. (c) DLCheXNet. (d) TDCheXNet. (e) LCheXNet. (f) LDCheXNet. (g) LDLCheXNet. (h) LTDCheXNet.

The intuitive display of pathological characteristics can clearly obtain each pathological area, and it can also measure the performance of a model in pathological detection. Figure 15 shows the display of salient features of pathological areas by different models.

It can be seen from Figure 15 that the upper left corner of each pathology display is the original image predicted by the input model, and the rest are the salient feature maps predicted by the 14 types of pathology. The input image is an image with Effusion pathology. The prediction results of CheXNet, DChXNet, DLChXNet, TDChXNet, LCheXNet, LDCheXNet, LDLCheXNet and LTDCheXNet are 85.65%, 84.64%, 88.66%, 94.26%, 83.48%, 90.94%, 85.12% and 89.54% respectively. However, TDChXNet achieved 94.26%. The presented results show that our improved method is effective, and the prediction indicators are higher than those of the other seven different models.

## 4 Discussion

We used an open‐source chest X‐ray dataset to improve the existing detection network, significantly addressing the low accuracy and speed of traditional pathology classification. Further research is needed to further enhance detection accuracy and inference speed and improve real‐time clinical diagnostic applications.

## 5 Conclusions

Pathological diagnosis of chest X‐ray images has always been a difficult problem for mankind. This paper proposes a new TDCheXNet network for chest X‐ray diagnosis and trains and tests it on the ChestX‐ray14 dataset. We improved upon the original CheXNet network. To reduce the loss of important information about pathological features during the downsampling process of the conversion layer, we removed the original conversion layer, used DSC and modified the first layer convolution, using the DSC structure to replace the original convolution kernel. Finally, for the corresponding convolution, we make full use of the pathological information in the negative direction of the x‐axis. The experimental results show that the model has good performance capabilities in chest X‐ray diagnosis.

## 6 Future Work

Currently, we propose the TDCheXNet network for chest X‐ray diagnosis. Fast and good diagnostic equipment has always been a weapon for radiologists to improve their work efficiency. How to improve this network to obtain a more lightweight model with higher detection accuracy is still a direction of our research. How to embed edge devices to achieve real‐time diagnosis is also a key research topic. We developed a new algorithm that can detect pathology in chest X‐ray images. Simple extensions of this algorithm can be used to detect a variety of diseases. By leveraging existing automated computer‐assisted technology, we hope this technology will improve healthcare delivery and increase diagnostic efficiency.

## Author Contributions

**Na Zhang:** conceptualization, methodology, validation, data curation, writing – review and editing, and visualization. **Guanghong Deng:** conceptualization, validation, formal analysis, and writing – original draft preparation. **Wenlong Jing:** resources, and funding acquisition. **Yong Li:** investigation, and supervision. **Li Li:** software, validation, and project administration.

## Consent

The authors have nothing to report.

## Conflicts of Interest

The authors declare no conflicts of interest.

## Data Integrity Statement

All authors have read and approved the final version of the manuscript. The corresponding author had full access to all of the data in this study and takes complete responsibility for the integrity of the data and the accuracy of the data analysis.

## Transparency Statement

The lead author Guanghong Deng, Wenlong Jing, Li Li affirms that this manuscript is an honest, accurate, and transparent account of the study being reported; that no important aspects of the study have been omitted; and that any discrepancies from the study as planned (and, if relevant, registered) have been explained.

## Data Availability

The dataset is a public open‐source dataset.
